# Infectious Diseases and Prematurity

**DOI:** 10.1155/2010/163046

**Published:** 2011-03-29

**Authors:** Bryan Larsen, Francesco DeSeta, Joseph Hwang, Mario Merialdi, José Tirán-Saucedo

**Affiliations:** ^1^College of Osteopathic Medicine, Marian University, Indianapolis, IN 46222, USA; ^2^University of Trieste, 34127 Trieste, Italy; ^3^Perinatal Center of Iowa and Mercy Medical Center, Des Moines, IA 50314, USA; ^4^Improving Maternal and Perinatal Health, FCH/RCR, World Health Organization, Switzerland; ^5^University of Monterrey, 66238 San Perdo Garía, NL, Mexico

Researchers and clinicians have for several decades focused epidemiologic, basic biomedical and clinical investigation on preterm birth, low birth weight, and numerous sequelae of untimely birth. As the problem has been examined from the expertise of various specialists and from separate but at times overlapping disciplines, different etiologic elements have emerged as important in the genesis of prematurity. Despite continuing discoveries in specific disciplines, preterm birth has not been etiologically tied to one factor such as genetic polymorphism, microbial challenge, or immune dysregulation. In recent years, the idea of “complex disease” has been proposed as a conceptual model for conditions that involve interplay between genetic, environmental, and other factors. This seems an apt description for preterm birth which will explain the breadth of topics in this special issue.

This special issue begins by considering microbial challenges to successful pregnancy. Certainly, the concept of infectious disease as a factor in preterm birth and its sequelae must consider the microorganisms that inhabit the normal host and the way in which host immune system interacts with these microbes. The first five contributions to this issue are focused primarily on microbes that can threaten the pregnancy. 

Foremost in understanding the microbial challenge to pregnancy is having the tools to appropriately characterize the maternal microbiome. In the paper by X. Zhou of Forney's laboratory, we are reminded that only since new, culture-independent, DNA-based methods of analysis have emerged we are able to correctly characterize the lower genital flora and establish that there is no longer one single list of microbes that can be called “normal.” These findings provide important clarity to concepts regarding flora and preterm birth. 

The second and third contributions in this series return to the problem represented by Group B *Streptococcus* (GBS). The concern for early identification and therapeutic intervention for GBS colonization in pregnancy has resulted in protocols that have become widely established. But experience over the past few decades has allowed a retrospective look at protocols again in specific clinical settings.

S. Faro et al. explore the experience of a private American hospital with respect to GBS screening and treatment. In 2 years of the study, they had an 89 percent screening rate with a 29 percent culture-positive rate with an invasive GBS incidence of 0.94 cases per 1000 live births. This is useful in underscoring the continuing need for vigilance for GBS. 

The third paper in this issue, also on the topic of GBS, looks at the issue with neonates exposed or presumptively exposed to the organism. B. Buckler and coworkers suggest that some workup of asymptomatic neonates “inadequately treated intrapartum” may be more costly and no better than clinical observation and treatment as warranted. Clearly, clinicians continue to seek refinements to their approaches to individual pathogens, and while the approach to GBS may be seen by some as firmly established, some details of the approach to this organism in pregnancy are open to continued review and assessment. 

Bacterial vaginosis is a topic that always emerges in discussions of preterm birth because of its epidemiologic association and the expansive literature that deals with the bacteriology and mechanistic associations with adverse pregnancy outcomes. Interest continues in finding antimicrobial approaches to the condition which in the case of the paper by I.-M. Bohbot and colleagues was aimed at clinical evaluation of secnidazole which they tested head to head with metronidazole. 

The fifth paper continues the description of specific organisms of concern in infectious conditions that may threaten pregnancy. The cell-wall deficient bacteria, *Mycoplasma* and *Ureaplasma*, are frequently mentioned as having relationships to adverse pregnancy outcome, but in reality, these organisms are rarely cultured routinely in clinical practice. J. Hwang and B. Larsen have provided an update on the current understanding of these organisms in pregnancy and have reviewed research that reveals the important role of these microbes in eliciting cytokine responses that can be contributors to preterm birth.

The cytokine responses to *Mycoplasma* and *Ureaplasma* provide a nice segue into the sixth paper by J. E. Thaxton and coworkers. A cogent and timely summary of some of the important immune response mediators that can influence premature labor and delivery is provided as the conceptual connection between microorganisms and the immune responses that can lead to preterm birth. More specifically, this report provides a compelling account of the role of toll-like receptor engagement with intrauterine bacteria and the possibility of a cytokine storm involved in prematurity. The possible existence of a second uterine inflammatory pathway is an additional intriguing concept raised by this paper.

As a continuum from term birth to late preterm birth, early preterm birth is one that may consider the most significant result of infectious complications of pregnancy to be the loss of the baby, and pregnancy loss is a problem of international importance with some 4.5 million stillbirths occurring annually worldwide. Thus, it is fitting that this issue includes a paper that recognizes this problem and offers some hopeful information. A cross-sectional study of plasmodial and helminthic infections in Ghana is reported by N. Yatich and coworkers who found a higher risk of stillbirth among pregnant women with parasitic infection and suggested that some pregnancies might be salvaged with early intervention if these conditions can be recognized earlier. 

The final paper in this issue by F. Omeaca et al. relates to the issues facing the preterm infant and one of the important threats to its survival and subsequent well-being. The authors explore the immune response of premature infants to hepatitis B vaccine as compared to term babies. In this report, while 6 of 94 premature infants did not respond, there were no significant differences in overall response to immunization due to low birthweight or gestational age suggesting the ability for vaccine application in this population.

The editors of this issue are pleased to offer paper that are not artificially focused but rather through the breadth of topics underscore the concept of complex disease mentioned at the beginning of this editorial. The complexity of genetics, immunity, and microbes acting in a tangled web of interactions reminds the reader why the issue of preterm birth has proven so intractable and why many different disciplines have something to contribute to the ultimate understanding of this clinical problem. 

But we are hopeful that as the reader considers these varied contributions, it will be appreciated that despite the clinical descriptions and scientific discoveries, there is an underlying fact that deserves remembering. The clinical and scientific verities involved in adverse pregnancy outcomes are very personally felt by families that lose babies to infection or who have to deal with infants who are severely compromised by their prematurity. These parents undoubtedly are hopeful that the pursuits of scientists and physicians will move ever closer to practical answers to this very prevalent and vexing problem that intrudes on the happiness that is otherwise part of the birth of a child. 

*Bryan Larsen* *Bryan Larsen* *Francesco DeSeta* *Francesco DeSeta* *Joseph Hwang* *Joseph Hwang* *Mario Merialdi* *Mario Merialdi* *José Tirán-Saucedo* *José Tirán-Saucedo*

